# Risk of seizures in a population of women with BRCA-positive metastatic breast cancer from an electronic health record database in the United States

**DOI:** 10.1186/s12885-023-10554-6

**Published:** 2023-01-24

**Authors:** Alexander Liede, Wendy Sebby, Ashok Kumar Reddy Miriyala, Ravi Potluri, Debasish Mazumder, Anirban Ghosh, Eros Papademetriou, Ryan Kilpatrick, Jerzy E. Tyczynski

**Affiliations:** 1 AbbVie Inc, North Chicago, IL USA; 2 AbbVie Limited, Dublin, Ireland; 3 Global Epidemiology, Pharmacovigilance & Patient Safety, AbbVie 14 Riverwalk, Citywest Business Campus, NSW D24 XN32 Dublin, Ireland; 4 SmartAnalyst Inc, New York, NY USA; 5 SmartAnalyst India Pvt. Ltd, Gurugram, India; 6 National Institute of Oncology, Warsaw, Poland

**Keywords:** Metastatic breast cancer, BRCA-positive, Seizures, Predisposing factors

## Abstract

**Background:**

Incidence and risk factors for seizures among women with advanced breast cancer (BC) and brain metastases are not well characterized across treatment-related or clinical subtypes. This study leveraged a large real-world dataset to describe incidence and risk factors for seizures in *BRCA*-associated metastatic breast cancer.

**Methods:**

The Optum® de-identified electronic health records database was used. Females with a BC diagnoses between 2008 and 2018, with clinic visits 12 months before BC index date, evidence of *BRCA* mutation (*BRCA*+), evidence of metastasis, and no previous cancers were included. Analyses were stratified by the overall *BRCA+* cohort and 4 molecular phenotypes: HER2+/HR- (human epidermal growth factor 2/hormone receptor), HER2−/HR+, HER2+/HR+, and triple negative BC (TNBC; HER2−/HR-). Seizures were identified using diagnosis codes and natural language processing. Incidence, occurrence rates, and cumulative incidence of seizures from the diagnosis of metastasis to the end of follow up were calculated. Comparisons were made between phenotypes and stratified on PARP inhibitor use, diagnosed brain metastases, history of seizures, and anticonvulsants use before BC. All comparisons included age at metastasis, number of prior lines of treatment, and metastasis location as covariates.

**Results:**

27.8% of 7941 *BRCA+* patients had ≥1 seizure over a mean follow-up time of 2.35 years. Incidence and occurrence rates were 11.83 (95% CI: 11.35–12.33) and 201.3 (95% CI: 198.05–204.50), respectively, per 100 person-years. HER2−/HR+ and TNBC patients had the lowest and highest seizure incidence rates, respectively (10.94 [95% CI: 10.23–11.71] and 16.83 [95% CI: 15.34–18.46]). With HER2−/HR+ as the reference group in a competing risk analysis, TNBC (hazard ratio, HR = 1.35; 95%CI: 1.21, 1.52; *p* < 0.001) and HER2+/HR- (HR = 1.29; 95%CI: 1.07, 1.56; *p* < 0.01) patients had a greater risk of seizures. Patients with diagnosed brain metastases or a history of seizures had higher seizure rates. Incidence trended higher with PARP inhibitor use, but patient numbers were low.

**Conclusions:**

This study provides novel real-world evidence on seizure incidence rates in *BRCA*+ BC patients, even those without diagnosed brain metastases, and underscores the need to understand patients’ tumor phenotypes when assessing seizure risk. These findings may have implications for clinical practice and assessment of benefit-risk ratios of new therapeutic agents.

**Supplementary Information:**

The online version contains supplementary material available at 10.1186/s12885-023-10554-6.

## Introduction

Breast cancer (BC) is the leading cancer type and ranks second among the causes of cancer-related mortality among women in the United States, with estimates indicating that 287,850 new cases will be diagnosed in 2022 and that 43,250 deaths will be attributable to it during the year [1]. Although advances in treatment have raised the five-year survival rate among women with localized BC at diagnosis to 99%, lower survival rates persist for women diagnosed with regional and particularly for women with distant disease (86 and 28%, respectively) [1].

While most breast tumors arise sporadically in the general population, a small subset (10–15%) are associated with genetic mutations, of which a majority (~ 60%) are associated with the *BRCA1* and *BRCA2* genes (*BR*east *CA*ncer susceptibility genes 1 and 2) [2–4]. Women who inherit a germline *BRCA* mutation are at a significantly increased risk for the development of breast cancer and ovarian cancer. Women face an approximately 72 and 69% risk of developing breast cancer associated with *BRCA1* and *BRCA2* mutations, respectively, by the age of 80, compared with 12% among women in the general population [3, 5]. Among *BRCA* mutations detected in women with breast cancer overall, approximately one-third may be somatic in origin [6, 7].

The three major molecular subtypes (or molecular phenotypes) of breast tumors based on the presence or absence of markers or overexpression of estrogen, progesterone (hormone) receptors (ER+ and/or PR+; collectively referred to as HR+), and the human epidermal growth factor 2 (HER2) are clinically meaningful to better characterize prognosis. Overall survival for women diagnosed with metastatic breast cancer also differs by molecular subtype and remains particularly low (10–13 months) for women with triple-negative tumors compared with the other molecular phenotypes (4–5 years) [8–11]. Accordingly, treatment guidelines for both non-metastatic and metastatic breast cancer are also specified by molecular subtype [8, 12]. Women diagnosed with metastatic breast cancer and HR+ phenotype receive endocrine therapy prior to the development of resistance to endocrine agents and single-agent chemotherapy thereafter, while patients with HER2+ tumors receive a combination of HER2+ targeting agents and chemotherapy in addition to endocrine therapy (the last only if the HER2+ tumors are also HR+) and patients with triple-negative tumors receive single-agent chemotherapy [8, 12]. Additional options available for the treatment of metastatic tumors harboring germline *BRCA* mutations include the poly [adenosine diphosphate-ribose] polymerase (PARP) inhibitors olaparib and talazoparib [13, 14]. These agents were approved in 2018 and are used in later lines of therapy for patients with HR+ and triple-negative tumors [8].

Organ sites associated with metastases from breast tumors include liver, bones, lungs, and/or brain. Diagnosed brain metastases in particular have been reported in 24% of breast cancer cases [15], with breast cancer ranking second among all causes of brain metastases [16]. The brain is the first site of metastasis in 12% of patients with breast cancer [17]. However, there is a paucity of data on rates of seizures among breast cancer patients with brain metastases and on potential risk factors associated with seizures in this patient population. Early reports on seizures in patients with brain metastases documented seizure frequencies ranging between 20 and 35% [18–20], but these studies included all cancer patients with brain metastases and not only breast cancer patients. A more recent review of 106 studies documented seizures in 12% of breast cancer patients with diagnosed brain metastases but did not explore the potential risk factors for seizures in these patients [15]. Other studies have reported agents used for cancer chemotherapy (e.g., methotrexate, fludarabine, cytarabine, vincristine, etoposide, and cisplatin) and other drugs prescribed to cancer patients (e.g., antidepressants such as tricyclics and bupropion, neuroleptic agents such as clozapine and phenothiazines, and antibiotics such as penicillin and β-lactams) as being epileptogenic [20–23]; however, no data are available on the incidence of seizures following the use of more recently approved therapeutic agents such as PARP inhibitors or on seizure rates categorized by molecular subtypes of breast cancer. This study was therefore undertaken with the objective of estimating, in a large real-world dataset, the incidence of seizures in patients with metastatic breast cancer who harbor mutations in the *BRCA1* or *BRCA2* genes, and examining factors associated with the development of seizures.

## Methods

### Data source

The Optum® de-identified electronic health records (EHR) database was used for this study [24]. The database represents an aggregation of patient-level data from more than 140,000 physicians at more than 700 hospitals and 7000 clinics that are part of 58 integrated delivery networks (IDNs) throughout the United States [25]. Extensive de-identified data on patient demographics, diagnoses, procedures, medications, laboratory results, and clinical administrative information for > 80 million patients from outpatient and inpatient settings, with > 7 million patients from each census region, are available within the database [25].

In addition to the structured data available from the EHR systems, natural language processing (NLP) was applied by Optum to the physicians’ notes to extract additional breast cancer data. The NLP step removed any identifiable information consistent with the Health Insurance Portability and Accountability Act (HIPAA) of 1996 [26], making Institutional Review Board (IRB) review and waiver unnecessary [27]. NLP technology assists in the extraction of information on signs, diseases, and symptoms (SDS) and data on biomarkers from physicians’ notes into structured data that is subsequently used to identify disease conditions without specific medical codes (e.g., ICD9/10 or HCPCS). The SDS terms are identified along with the sentiment associated with the term. For example, to identify a patient who may have had a seizure, the SDS term “seizure” may exist in the patient’s record, but if the SDS attribute is “does not have”, it is interpreted as a physician writing in their notes that the patient does not have seizures. *BRCA* mutation-positive status was determined by NLP-related data fields. While the data source indicated the presence of *BRCA* mutations, it did not provide information on whether the mutations were germline or somatic in origin.

The Optum EHR database was selected primarily due to the number of patients available and the NLP extracted biomarker and seizure information. In addition, the Optum EHR database, is general (i.e. not oncology center specific), which allowed us to include a wider variety of treating physicians.

### Inclusion criteria

Female patients with BC ICD9/10 diagnosis (ICD-9 code 174.xx or ICD-10 codes C50.01, C50.11, C50.21, C50.31, C50.41, C50.51, C50.61, C50.81, and C50.91) between January 1, 2008, and December 31, 2018, were included if they were ≥ 18 years of age on the BC index date (first BC diagnosis in the database), had evidence of EHR activity at least 12 months prior to the BC index date, had no other primary or secondary cancers 12 months prior to the BC index date, had ≥1 diagnosis or SDS term indicating metastasis more than 30 days prior to the BC index date, and had evidence of being *BRCA+*. The diagnosis codes used to identify patients with malignancies and the SDS terms/attributes used to identify patients with metastases but without ICD codes are listed in Additional file 1 Supplementary Table 1 and Additional file 2 Supplementary Table 2, respectively.

### Analyses performed

The analyses described below were performed both for the overall *BRCA+* cohort and stratified by four phenotypes: HER2+/HR+, HER2+/HR-, HER2−/HR+, and TNBC (triple negative breast cancer: HER2− and HR−). Seizures were identified using diagnosis codes (ICD-9: 345.xx [epilepsy and recurrent seizures] and 780.39 [other convulsions]; ICD-10: G40.xx [epilepsy and recurrent seizures] and R56.9 [unspecified convulsions]) and the SDS terms listed in Additional file 3 Supplementary Table 3.

The rates of occurrence and incidence of seizures in the post-metastasis period (defined as the time between the date of diagnosis of metastases and the end of follow up) were calculated for each patient and the cumulative incidence curves for drug exposures or follow up from diagnoses of brain metastases were plotted. The occurrence rate was defined as the total or aggregate number of days with a seizure divided by the aggregate duration in the post-metastasis period for patients with a seizure. The seizure incidence rate was calculated using a Poisson model where the follow-up period was defined as the date of metastasis to the date of the first seizure (for those with a seizure) or the date of metastasis to the end of follow-up (for those without a seizure). Incidence rates for the four phenotypes are presented as Forest plots.

Incidence rates expressed in units of per person-time and adjusted for age at metastasis, number of prior lines of treatment, and metastasis location were calculated, with comparisons made between patients with and without the following risk factors: use of PARP inhibitors, diagnosed brain metastases, history of seizures prior to the breast cancer diagnosis, and use of anticonvulsants prior to the diagnosis of breast cancer (see Additional file 4 Supplementary Table 4 for list of anticonvulsants and PARP inhibitors). Competing risks regression of time to seizure, with death as a competing event, and the same covariates as above were performed overall and for the four risk factors. Cumulative incidence curves of time to seizure were plotted for the overall sample and the four risk factors. These plots were stratified by the four phenotypes and a Grey’s test of equality performed to test for overall differences between the curves.

## Results

### Patient identification and demographics

**Fig. 1:**
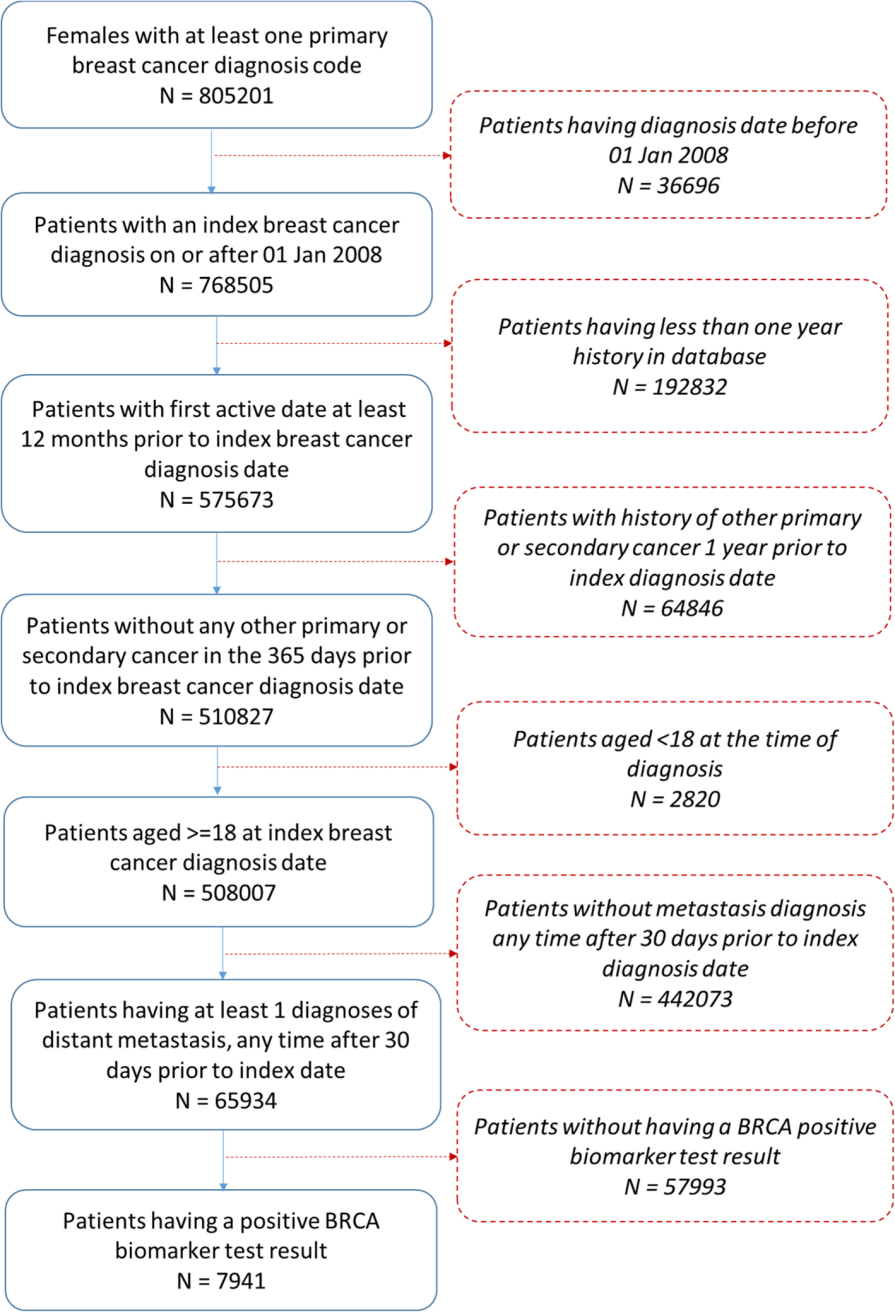
Patient attrition

A total of 7941 *BRCA+* breast cancer patients were identified in the Optum® dataset for the study period from among 65,934 (12.0%) metastatic breast cancer patients (Fig. 1).

Of these, 5922 patients had known HR and HER2 status, among whom 1323 (22.3%) patients had the triple negative phenotype (ER−, PR−, and HER2−); 1039 (17.5%) patients had the HER2+/HR+ phenotype, 378 (6.4%) patients had the HER2+/HR− phenotype, and 3182 (53.7%) patients had the HER2−/HR+ phenotype (Table 1).

**Table 1:** Patient demographics in BRCA+ study cohort by seizure occurrence

	Overall		With seizures		Without seizures	
	***n***	%	***n***	%	***n***	%
	7941	100%	2207	28%	5734	72%
**Race**						
Caucasian	6454	81.3%	1783	80.8%	4671	81.5%
African American	849	10.7%	273	12.4%	576	10.0%
Asian	165	2.1%	39	1.8%	126	2.2%
Other/Unknown	473	6.0%	112	5.1%	361	6.3%
**Ethnicity**						
Non-Hispanic	7292	91.8%	2050	92.9%	5242	91.4%
Hispanic	308	3.9%	77	3.5%	231	4.0%
Unknown	341	4.3%	80	3.6%	261	4.6%
**Region**						
Midwest	4065	51.2%	1294	58.6%	2771	48.3%
South	1513	19.1%	374	16.9%	1139	19.9%
West	888	11.2%	172	7.8%	716	12.5%
Northeast	1239	15.6%	285	12.9%	954	16.6%
Other/Unknown	236	3.0%	82	3.7%	154	2.7%
**Age at diagnosis (years)**						
Mean (SD)	53.6 (12.74)		53.6 (12.81)		53.6 (12.71)	
Median (IQR)	53 (44–62)		53 (44–62)		53 (44–62)	
Min - Max	18–89		19–88		18–89	
< 65 years of age	6308	79.4%	1753	79.4%	4555	79.4%
≥ 65 years of age	1633	20.6%	454	20.6%	1179	20.6%
Hormonal Status* (*n* **= 5922)**						
HER2+/HR+	1039	17.5%	298	5.0%	741	12.5%
HER2+/HR-	378	6.4%	124	2.1%	254	4.3%
HER2−/HR+	3182	53.7%	848	14.3%	2334	39.4%
TNBC	1323	22.3%	442	7.5%	881	14.9%

*Of those with a known status; HER2 = human epidermal growth factor receptor 2; HR = hormone receptor; TNBC = triple negative breast cancer

The majority of the patients were Caucasians (81.3%) while geographically, most patients were based in the Midwest (51.2%). The mean age (standard deviation) of patients was 53.6 (12.7) years; this did not differ between patients with and without seizures. The majority (79.4%) of the patients were < 65 years of age; percentages of patients below and above 65 years of age did not differ between patients with and without seizures.

### Incidence rates, overall and by phenotype

**Fig. 2:**
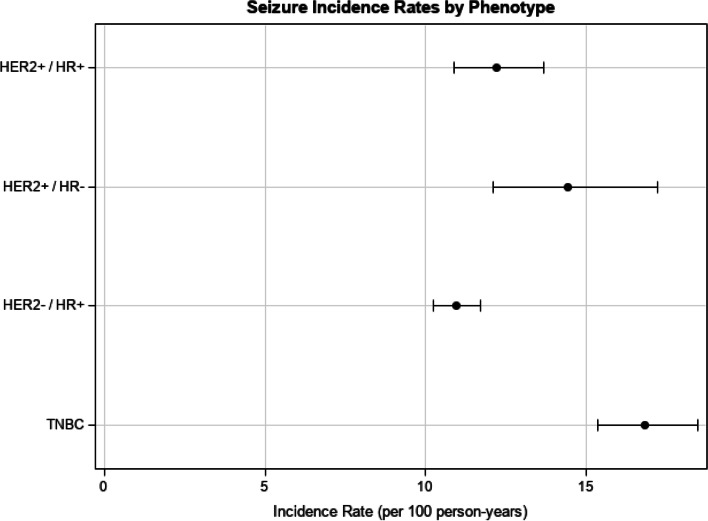
Seizure incidence rates in BRCA+ patients overall and by HER2 and HR phenotype

Of the 7941 patients in the overall cohort, 2207 (27.8%) had at least one seizure, with an incidence rate of 11.83 (95% CI: 11.35, 12.33) per 100 person-years and the occurrence rate of 201.3 (95% CI: 198.05, 204.50) per 100 person-years (Fig. 2 and Table 2).

**Table 2:** Seizure incidence and occurrence rates in BRCA+ patients overall and by phenotype

	***N***	Number of patients with seizures	Median follow-up duration to seizure (days)	% patients with at least one seizure	Incidence person-years*	Incidence rate** (95% CI)	Total Occurrences	Occurrence frequency	Occurrence person-years^**†**^	Occurrence rate**
Overall	7941	2207	161	27.80%	18655	11.83 (11.35,12.33)	15029	6.80	7468	201.30
HER2+/HR+	1039	298	193.5	28.68%	2443	12.20 (10.89,13.66)	2492	8.36	1051	237.13
HER2+/HR-	378	124	176.5	32.80%	860	14.43 (12.10,17.20)	1191	9.60	471	252.74
HER2−/HR+	3182	848	147	26.65%	7749	10.94 (10.23,11.71)	5212	6.15	2917	178.66
HER2−/HR-	1340	447	149	33.36%	2656	16.83 (15.34,18.46)	3171	7.09	1312	241.72

HER2 = human epidermal growth factor receptor 2; HR = hormone receptor; TNBC = triple negative breast cancer

*Date of metastasis to first seizure date or end of follow-up for those without a seizure

**Per 100 person-years

†Date of metastasis to the end of follow-up among patients with a seizure

Among patients with seizures, there were no meaningful demographic differences between phenotype subgroups. Among phenotypes, patients in the HER2−/HR+ subgroup had the lowest seizure incidence rate of 10.94 per 100 person-years (95% CI: 10.23, 11.71), while patients with the TNBC phenotype had the highest seizure incidence rate of 16.83 per 100 person-years (95% CI: 15.34, 18.46) (Table 2).

### Incidence rates by risk factors

Overall, BRCA+ patients with diagnosed brain metastases or a history of seizures had higher seizure incidence rates than those without the respective risk factors (Table 3). Prior use of anticonvulsants did not affect the seizure incidence rate. While the number of patients using PARP inhibitors is too low to draw meaningful conclusions, the incidence rates trend higher for those using PARP inhibitors versus those that do not use them.

**Table 3:** Seizure incidence and occurrence rates for all BRCA+ patients (adjusted for age at metastasis, number of prior lines of treatment, and metastasis location)

	***N***	***N*** patients with a seizure	Median duration to seizure (days)	% patients with at least one seizure	Incidence person-years*	Incidence rate** (95% CI)	Total Occurrences	Occurrence frequency	Occurrence person-years†	Occurrence rate**
**Overall**	7941	2207	161	27.8%	18655	7.28 (6.01–8.83)	15,029	6.81	7468	201.26
**Use of PARP inhibitors during evaluation period**										
Patients exposed to PARP inhibitors	92	16	53.5	17.4%	61	76.58 (7.81–751.26)	93	5.81	20	458.04
Patients not exposed to PARP inhibitors	7849	2175	161	27.7%	18452	7.20 (5.94–8.74)	14827	6.82	7379	200.93
**Diagnosed brain metastases during evaluation period**										
Patients with diagnosed brain metastases	1041	504	9	48.4%	1002	42.55 (28.59–63.30)	4071	8.08	951	428.06
Patients without diagnosed brain metastases	6900	1627	153	23.6%	16874	5.26 (4.20–6.59)	9994	6.14	5796	172.43
**History of seizures prior to index date**										
Patients with history of seizures	788	465	22	59.0%	913	34.76 (23.24–52.01)	5532	11.90	1336	414.12
Patients without history of seizures	7153	1742	237.5	24.4%	17743	6.35 (5.11–7.88)	9497	5.45	6132	154.88
**Use of anticonvulsants prior to index date**										
Patients with history anticonvulsants	2280	688	122.5	30.2%	5057	7.6 (5.38–10.73)	5956	8.66	2250	264.67
Patients without history of anticonvulsants	5661	1519	178	26.8%	13598	6.93 (5.50–8.75)	9073	5.97	5217	173.91

*Follow-up duration is from the date of metastasis to first seizure date or end of follow-up for those without a seizure. Duration is in person-years

**Per 100 person-years

†Follow-up duration is from the date of metastasis to the end of follow-up among patients with a seizure

### Time to seizure

**Fig. 3:**
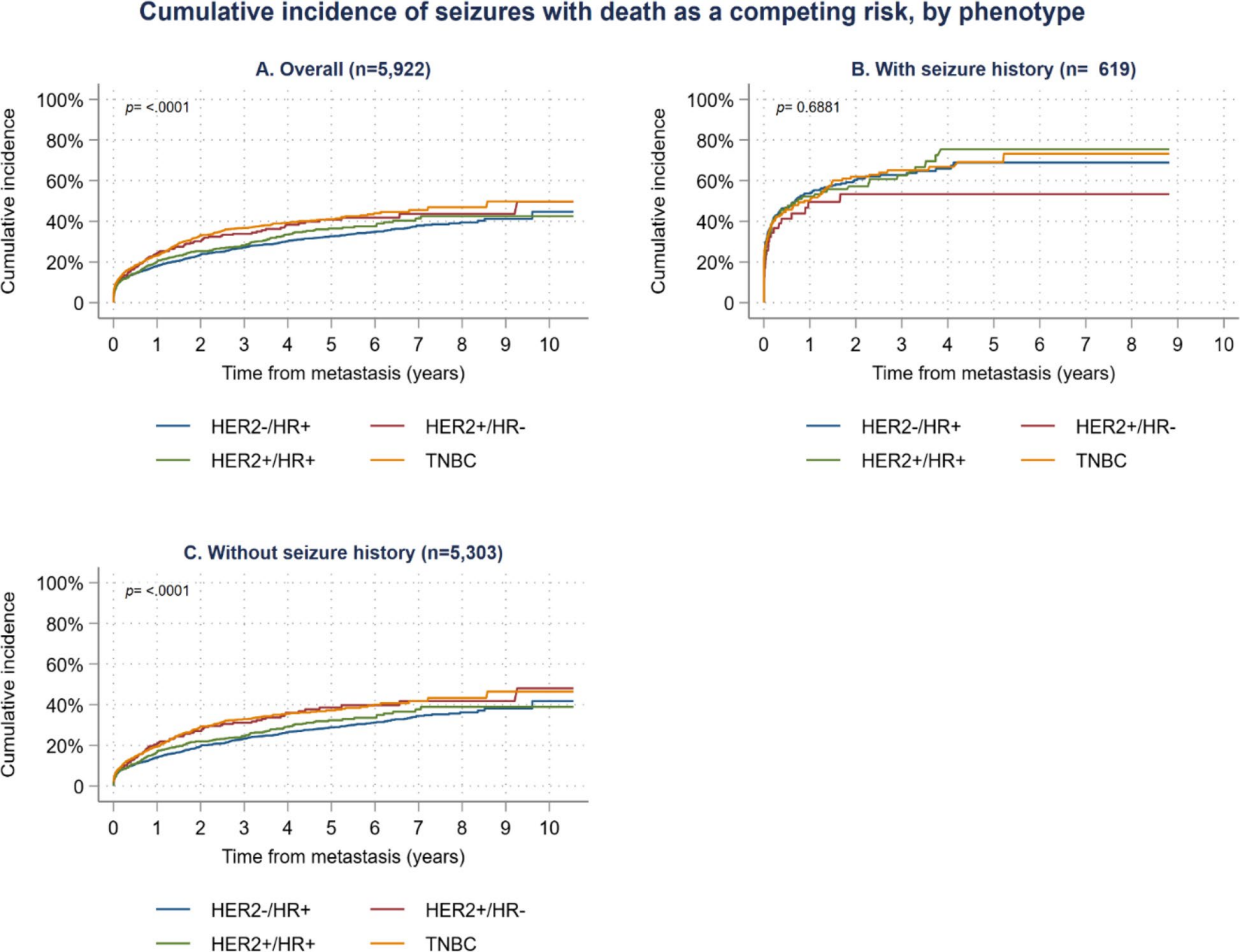
Time to seizure in BRCA+ patients by HER2/HR phenotype, overall and by prior seizure status

Cumulative incidence plots showed differences in the time to a seizure between the four phenotypes (*p* < 0.001, Fig. 3a). At 2 years, the cumulative incidence of a seizure was 33.1% for TNBC patients, 30.3% for HER2+/HR- patients, 25.4% for HER2+/HR+ patients, and 23.6% for HER2−/HR+ patients. At 8 years, these rates increased to 46.9, 43.7, 42.5, 39.5% respectively. Competing risks regression showed that overall, patients with the TNBC (HR = 1.35; 95%CI: 1.21, 1.52; p < 0.001) and HER2+/HR- (HR = 1.29; 95%CI: 1.07, 1.56; *p* < 0.01) phenotypes had increased risk of a seizure compared to the HER2−/HR+ phenotype (Table 4). For patients with a history of seizures, rates of seizures were higher, but not different between the phenotypes (*p* = 0.687, Fig. 3b). At 2 years, the cumulative incidence of a seizure was between 61.0 and 75.4% depending on their phenotype and at 8 years, it was between 53.4 and 75.4%. For patients without a history of seizures, the cumulative incidence of a seizure was 29.1% for TNBC patients, 27.1% for HER2+/HR- patients, 22.0% for HER2+/HR+ patients, and 19.6% for HER2−/HR+ patients. At 8 years, these rates increased to 43.3, 41.8, 38.9, 36.2% respectively (*p* < 0.001, Fig. 3c). Competing risks regression showed that in those without a history of seizures, patients with the TNBC phenotype had a significant hazard ratio of 1.40 (95%CI: 1.23, 1.60; p < 0.001) and the HER2+/HR- phenotype had significant hazard ratio of 1.38 (95%CI: 1.12, 1.71; *p* < 0.01) compared to the HER2−/HR+ phenotype (Table 4).

**Table 4:** Competing risks regression of seizures by phenotype and with/without seizure history

Phenotype	Overall (***n*** = 5911)		With seizure history (***n*** = 616)		Without seizure history (***n*** = 5295)		History of anticonvulsant use (***n*** = 1690)		History of brain metastasis (***n*** = 818)	
	HR	95% CI	HR	95% CI	HR	95% CI	HR	95% CI	HR	95% CI
HER2−/HR+	1.00	Ref.	1.00	Ref.	1.00	Ref.	1.00	Ref.	1.00	Ref.
HER2+/HR-	1.29**	(1.07, 1.56)	0.75	(0.49, 1.16)	1.38**	(1.12, 1.71)	1.45*	(1.07, 1.96)	1.01	(0.79, 1.30)
HER2+/HR+	1.10	(0.97, 1.26)	1.00	(0.77, 1.31)	1.12	(0.97, 1.30)	1.05	(0.82, 1.33)	1.04	(0.90, 1.22)
TNBC	1.35***	(1.21, 1.52)	0.99	(0.78, 1.26)	1.40***	(1.23, 1.60)	1.05	(0.85, 1.30)	1.12	(0.97, 1.29)

Note: Adjusted for age at metastasis, number of prior lines of therapy, and site of metastasis

* *p* < 0.05, ** *p* < 0.01, *** *p* < 0.001

**Fig. 4:**
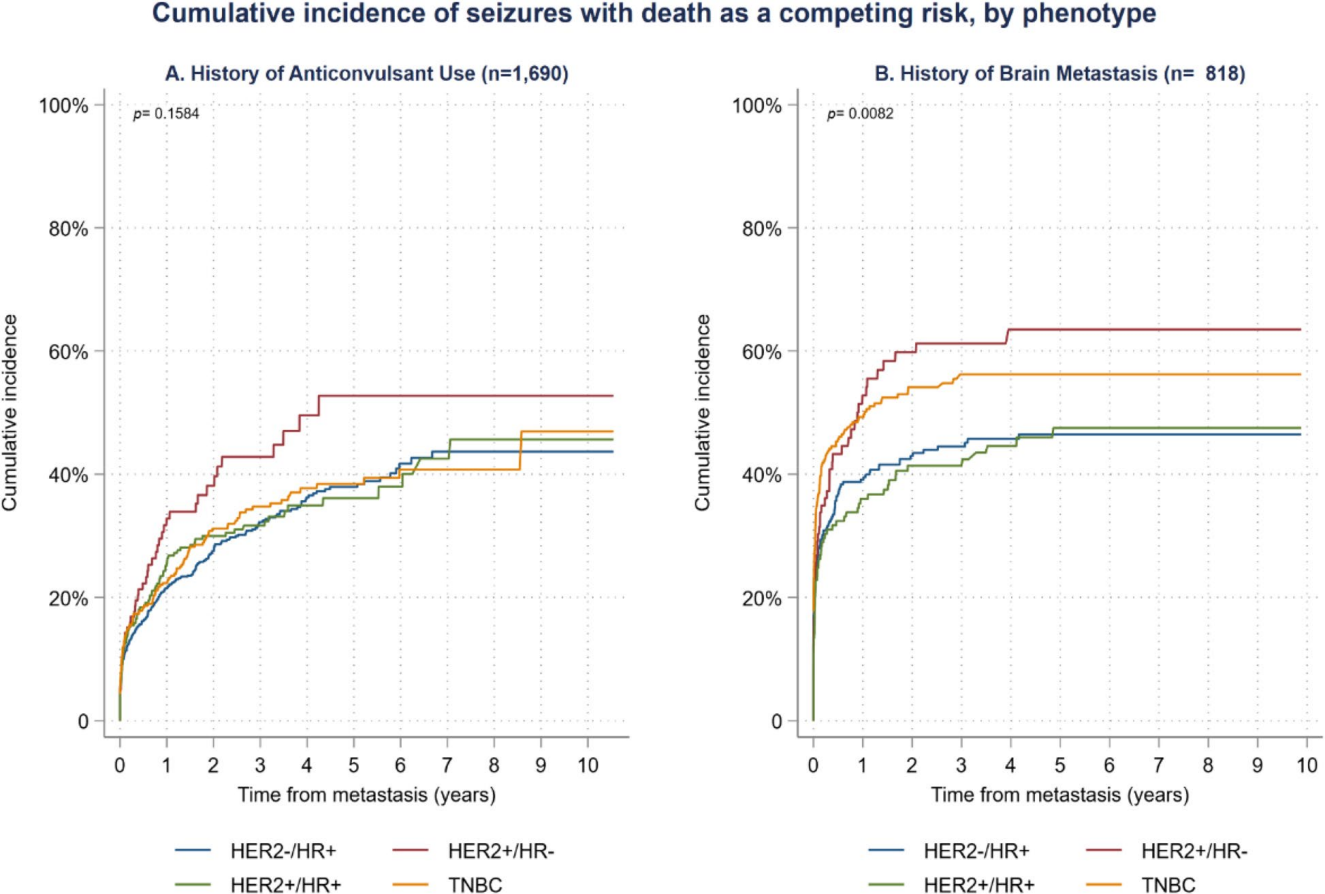
Time to seizure in BRCA+ patients by phenotype and presence of anticonvulsant use or brain metastasis

For patients with a history of anticonvulsant use, only HER2+/HR- patients showed an increased risk of seizure (HR = 1.45; 95% CI: 1.07, 1.96 l; *p* < 0.05; Fig. 4a). In patients with diagnosed brain metastases, both TNBC (HR = 1.39; 95%CI: 1.09, 1.76; p < 0.01) and HER2+/HR- (HR = 1.38; 95%CI: 1.02, 1.88; p < 0.05) phenotypes showed an increased risk of seizure (Fig. 4b). There was no statistically significant difference in time to seizure between the phenotypes among patients a history of using PARP inhibitors (data not shown).

## Discussion

Data pertaining to the risk of seizures among women with advanced or metastatic breast cancer is limited, particularly when considering women who may harbor a mutation in the high risk *BRCA1* and *BRCA2* breast cancer predisposition genes. This study identified a large cohort of 7941 women with *BRCA*-associated metastatic breast cancer treated at centers across the United States as represented in a large EHR database and estimated the overall incidence rate of seizures to be 11.83 (95% CI: 11.35,12.33) per 100 patient-years after index diagnosis of metastatic disease.

The group of women with the highest seizure incidence rates of 16.83 events (95% CI: 15.34, 18.46) per 100 patient-years were those affected with triple-negative breast cancer. Additional risk groups were women with diagnosed brain metastases (incidence rate = 42.55 [95% CI: 28.59, 63.30] per 100 patient-years), and women with a history of seizures (incidence rate = 34.76 [95% CI: 23.24, 52.01] per 100 patient-years.

Overall, patients with brain metastasis or a history of seizures had higher seizure incidence rates, which is consistent with clinical impressions and previous studies [15]. TNBC and HER2−/HR+ patients showed a higher risk of developing seizures over the course of the study. Patients with these phenotypes were also at higher risk when metastasis was in the brain and if they had a history of anticonvulsant use. Although our study was limited in the sample size available for analyses of PARP inhibitor-treated patients, seizure incidence rates in this patient population appeared to be higher than in patients who were not treated with PARP inhibitors (76.58 per 100 person-years vs 7.20 per 100 person-years).

A notable strength of this study was the use of unstructured NLP fields in addition to the structured data (ICD9/10 diagnosis codes) to identify the seizure outcome. In a prior study, we showed how the combination of structured and unstructured data to identify adverse event outcomes is superior to using structured data alone [28].

Although *BRCA* mutation-positive status was discernable in the EHR data from unstructured NLP fields, a limitation of our study was the incomplete available information related to *BRCA1* and *BRCA2* status. Specifically, 5383 (67.8%) patients did not have details on which gene (*BRCA1* versus *BRCA2*) was implicated. Furthermore, the Optum® EHR database did not provide differentiation on whether the *BRCA* mutation was a somatic or germline variant. Further, while large, we used a single EHR database for this study. This may introduce biases in terms of types of patients (e.g. the high proportion of Caucasian and Midwestern patients) and the types of treating physicians. The database does not contain information regarding healthcare access, lifestyle, or socio-economic status, which may also introduce bias into our analyses.

This study provides novel real-world evidence on the incidence rates of seizures affecting a large population of women with metastatic *BRCA*-associated breast cancer who received care in clinics across the U.S. The study highlights the importance of understanding patients’ molecular subtypes associated with breast cancer when assessing seizure risk. The seizure incidence rate was highest in the subgroup of women with TNBC, and significantly higher for women with diagnosed brain metastases, with a history of seizures/anti-convulsive therapy, as well as those receiving PARP inhibitor therapy. These findings have implications for clinical practice as well as for drug development when considering the benefit-risk of new oncologic therapeutic agents (such as PARP inhibitors studied here) that, once approved, are mainly introduced for treating patients with advanced disease (distant metastases) who have failed several lines of therapy. Further work may characterize seizure risk across all stages of *BRCA*-associated breast cancer.

## Supplementary Information

**Additional file 1: Supplementary Table 1.** Diagnosis codes used to identify patients with malignancies.

**Additional file 2: Supplementary Table 2.** SDS information used to identify patients with metastases but without ICD codes in their EHRs.

**Additional file 3: Supplementary Table 3.** SDS terms used to identify patients with seizures.

**Additional file 4: Supplementary Table 4.** List of anticonvulsants and PARP inhibitors included in the analyses.

## Data Availability

The datasets generated during and/or analysed during the current study are not publicly available due to the proprietary nature of the database from which they were derived and used under license for the current study. However, the data are available from the corresponding author on reasonable request and with permission of Optum®.

## Abbreviations

BC: Breast cancer
*BRCA1*/*BRCA2*: BReast CAncer genes 1 and 2
CI: Confidence intervals
EHR: Electronic health record
ER: Estrogen receptor
HCPCS: Healthcare Common Procedural Coding System
HER2: Human epidermal growth factor 2
HIPAA: Health Insurance Portability and Accountability Act
ICD: International Statistical Classification of Diseases and Related Health Problems
IDN: Integrated delivery network
IQR: Interquartile range
IRB: Institutional Review Board
NLP: Natural Language Processing
PARP: Poly [adenosine diphosphate-ribose] polymerase
PR: Progesterone receptor
SD: Standard deviation
SDS: Signs, diseases, and symptoms
TNBC: Triple-negative breast cancer.
