# Supplementary material for: Risk of seizures in a population of women with BRCA-positive metastatic breast cancer from an electronic health record database in the United States
Source: BMC Cancer. 2023 Jan 24;23:78. doi: 10.1186/s12885-023-10554-6 (PMC9872301; doi:10.1186/s12885-023-10554-6)
Supplement: Supplementary file 1 — Additional file 1: Supplementary Table 1. Diagnosis codes used to identify patients with malignancies. [file 12885_2023_10554_MOESM1_ESM.docx]

**Supplementary Table 1.** Diagnosis codes used to identify patients with malignancies.

| **Code** | **Description** | **ICD Version** |
| --- | --- | --- |
| 1960 | Secondary and Unspecified Malignant Neoplasm of Lymph Nodes of Head, Face, and Neck | ICD-09 |
| 1961 | Secondary and Unspecified Malignant Neoplasm of Intrathoracic Lymph Nodes | ICD-09 |
| 1962 | Secondary and Unspecified Malignant Neoplasm of Intra-Abdominal Lymph Nodes | ICD-09 |
| 1965 | Secondary and Unspecified Malignant Neoplasm of Lymph Nodes of Inguinal Region and Lower Limb | ICD-09 |
| 1966 | Secondary and Unspecified Malignant Neoplasm of Intrapelvic Lymph Nodes | ICD-09 |
| 1968 | Secondary and Unspecified Malignant Neoplasm of Lymph Nodes of Multiple Sites | ICD-09 |
| 1969 | Secondary and Unspecified Malignant Neoplasm of Lymph Nodes, Site Unspecified | ICD-09 |
| 1970 | Secondary Malignant Neoplasm of Lung | ICD-09 |
| 1971 | Secondary Malignant Neoplasm of Mediastinum | ICD-09 |
| 1972 | Secondary Malignant Neoplasm of Pleura | ICD-09 |
| 1973 | Secondary Malignant Neoplasm of Other Respiratory Organs | ICD-09 |
| 1974 | Secondary Malignant Neoplasm of Small Intestine Including Duodenum | ICD-09 |
| 1975 | Secondary Malignant Neoplasm of Large Intestine and Rectum | ICD-09 |
| 1976 | Secondary Malignant Neoplasm of Retroperitoneum and Peritoneum | ICD-09 |
| 1977 | Malignant Neoplasm of Liver, Secondary | ICD-09 |
| 1978 | Secondary Malignant Neoplasm of Other Digestive Organs and Spleen | ICD-09 |
| 1980 | Secondary Malignant Neoplasm of Kidney | ICD-09 |
| 1981 | Secondary Malignant Neoplasm of Other Urinary Organs | ICD-09 |
| 1982 | Secondary Malignant Neoplasm of Skin | ICD-09 |
| 1983 | Secondary Malignant Neoplasm of Brain and Spinal Cord | ICD-09 |
| 1984 | Secondary Malignant Neoplasm of Other Parts of Nervous System | ICD-09 |
| 1985 | Secondary Malignant Neoplasm of Bone and Bone Marrow | ICD-09 |
| 1986 | Secondary Malignant Neoplasm of Ovary | ICD-09 |
| 1987 | Secondary Malignant Neoplasm of Adrenal Gland | ICD-09 |
| 19882 | Secondary Malignant Neoplasm of Genital Organs | ICD-09 |
| 19889 | Secondary Malignant Neoplasm of Other Specified Sites | ICD-09 |
| C77 | Secondary and unspecified malignant neoplasm of lymph nodes | ICD-10 |
| C770 | Secondary and unspecified malignant neoplasm of lymph nodes of head, face, and neck | ICD-10 |
| C771 | Secondary and unspecified malignant neoplasm of intrathoracic lymph nodes | ICD-10 |
| C772 | Secondary and unspecified malignant neoplasm of intra-abdominal lymph nodes | ICD-10 |
| C774 | Secondary and unspecified malignant neoplasm of inguinal and lower limb lymph nodes | ICD-10 |
| C775 | Secondary and unspecified malignant neoplasm of intrapelvic lymph nodes | ICD-10 |
| C778 | Secondary and unspecified malignant neoplasm of lymph nodes of multiple regions | ICD-10 |
| C779 | Secondary and unspecified malignant neoplasm of lymph node, unspecified | ICD-10 |
| C78 | Secondary malignant neoplasm of respiratory and digestive organs | ICD-10 |
| C780 | Secondary malignant neoplasm of lung | ICD-10 |
| C7800 | Secondary malignant neoplasm of unspecified lung | ICD-10 |
| C7801 | Secondary malignant neoplasm of right lung | ICD-10 |
| C7802 | Secondary malignant neoplasm of left lung | ICD-10 |
| C781 | Secondary malignant neoplasm of mediastinum | ICD-10 |
| C782 | Secondary malignant neoplasm of pleura | ICD-10 |
| C783 | Secondary malignant neoplasm of other and unspecified respiratory organs | ICD-10 |
| C7830 | Secondary malignant neoplasm of unspecified respiratory organ | ICD-10 |
| C7839 | Secondary malignant neoplasm of other respiratory organs | ICD-10 |
| C784 | Secondary malignant neoplasm of small intestine | ICD-10 |
| C785 | Secondary malignant neoplasm of large intestine and rectum | ICD-10 |
| C786 | Secondary malignant neoplasm of retroperitoneum and peritoneum | ICD-10 |
| C787 | Secondary malignant neoplasm of liver and intrahepatic bile duct | ICD-10 |
| C788 | Secondary malignant neoplasm of other and unspecified digestive organs | ICD-10 |
| C7880 | Secondary malignant neoplasm of unspecified digestive organ | ICD-10 |
| C7889 | Secondary malignant neoplasm of other digestive organs | ICD-10 |
| C79 | Secondary malignant neoplasm of other and unspecified sites | ICD-10 |
| C790 | Secondary malignant neoplasm of kidney and renal pelvis | ICD-10 |
| C7900 | Secondary malignant neoplasm of unspecified kidney and renal pelvis | ICD-10 |
| C7901 | Secondary malignant neoplasm of right kidney and renal pelvis | ICD-10 |
| C7902 | Secondary malignant neoplasm of left kidney and renal pelvis | ICD-10 |
| C791 | Secondary malignant neoplasm of bladder and other and unspecified urinary organs | ICD-10 |
| C7910 | Secondary malignant neoplasm of unspecified urinary organs | ICD-10 |
| C7911 | Secondary malignant neoplasm of bladder | ICD-10 |
| C7919 | Secondary malignant neoplasm of other urinary organs | ICD-10 |
| C792 | Secondary malignant neoplasm of skin | ICD-10 |
| C793 | Secondary malignant neoplasm of brain and cerebral meninges | ICD-10 |
| C7931 | Secondary malignant neoplasm of brain | ICD-10 |
| C7932 | Secondary malignant neoplasm of cerebral meninges | ICD-10 |
| C794 | Secondary malignant neoplasm of other and unspecified parts of nervous system | ICD-10 |
| C7940 | Secondary malignant neoplasm of unspecified part of nervous system | ICD-10 |
| C7949 | Secondary malignant neoplasm of other parts of nervous system | ICD-10 |
| C795 | Secondary malignant neoplasm of bone and bone marrow | ICD-10 |
| C7951 | Secondary malignant neoplasm of bone | ICD-10 |
| C7952 | Secondary malignant neoplasm of bone marrow | ICD-10 |
| C796 | Secondary malignant neoplasm of ovary | ICD-10 |
| C7960 | Secondary malignant neoplasm of unspecified ovary | ICD-10 |
| C7961 | Secondary malignant neoplasm of right ovary | ICD-10 |
| C7962 | Secondary malignant neoplasm of left ovary | ICD-10 |
| C797 | Secondary malignant neoplasm of adrenal gland | ICD-10 |
| C7970 | Secondary malignant neoplasm of unspecified adrenal gland | ICD-10 |
| C7971 | Secondary malignant neoplasm of right adrenal gland | ICD-10 |
| C7972 | Secondary malignant neoplasm of left adrenal gland | ICD-10 |
| C798 | Secondary malignant neoplasm of other specified sites | ICD-10 |
| C7982 | Secondary malignant neoplasm of genital organs | ICD-10 |
| C7989 | Secondary malignant neoplasm of other specified sites | ICD-10 |
| C799 | Secondary malignant neoplasm of unspecified site | ICD-10 |
