# Supplementary material for: Risk of seizures in a population of women with BRCA-positive metastatic breast cancer from an electronic health record database in the United States
Source: BMC Cancer. 2023 Jan 24;23:78. doi: 10.1186/s12885-023-10554-6 (PMC9872301; doi:10.1186/s12885-023-10554-6)
Supplement: Supplementary file 2 — Additional file 2: Supplementary Table 2. SDS information used to identify patients with metastases but without ICD codes in their EHRs. [file 12885_2023_10554_MOESM2_ESM.docx]

**Supplementary Table 2.** SDS information used to identify patients with metastases but without ICD codes in their EHRs.

1. Stage determination based on availability of stage-related terms.

| **Stage** | **Terms** |
| --- | --- |
| Metastasis | Stage IV |
|  | End stage |
|  | Advanced stage |
|  | Metastatic |
|  | Late-stage |

1. Metastasis determination using TNM information in the SDS dataset.

| **Stage** | **T** | **N** | **M** |
| --- | --- | --- | --- |
| Metastasis | Any T | Any N | M1a-b |
|  | Any T | Any N | M1c |

1. SDS search strings used in the NLP algorithm.

| **SDS_TERM** | **SDS_ATTRIBUTE** | **SDS_SENTIMENT** |
| --- | --- | --- |
| cancer | metastatic |  |
| malignant neoplasm | stage=4 | diagnose |
| carcinoma | stage=4^metastatic | have |
| ductal carcinoma | recurrent; metastatic | exhibit |
| lump | advanced | present |
| adenocarcinoma | stage=4^invasive | positive |
| dcis | metastatic; invasive |  |
| ductal carcinoma in situ | progressive; metastatic |  |
| malignancy | metastatic; lobular |  |
| lobular carcinoma | metastatic; inflammatory |  |
| idc | metastasized |  |
| redness | stage=4^inflammatory |  |
| metastasis | likely; metastatic |  |
| ductal cancer | advanced; metastatic |  |
| lobular carcinoma in situ | metastatic; recurrent |  |
| nipple discharge | stage=4^infiltrating |  |
| lcis | probable; metastatic |  |
| dimpling | stage=4^recurrent |  |
| idca | metastatic; infiltrating |  |
| Paget’s disease | widespread; metastatic |  |
| cancers | metastatic; unspecified |  |
| lobular cancer | biopsy-proven; metastatic |  |
| retraction | malignant; metastatic |  |
| angiosarcoma | extensive; metastatic |  |
| inverted nipple | end-stage |  |
| metastatic disease | advanced-stage |  |
| ulceration | long-standing; metastatic |  |
| sarcoma | metastatic; poorly differentiated |  |
| ulcer | stage=4^unspecified |  |
| bloody nipple discharge | significant; metastatic |  |
| basal cell carcinoma | end-stage; metastatic |  |
| invasion | oligometastatic |  |
| squamous cell carcinoma | metastatic; bilateral |  |
| ulcerated mass | active; metastatic |  |
| ulcers | diffusely metastatic |  |
| breast discharge | diffuse; metastatic |  |
| carcinosarcoma | hormone refractory; metastatic |  |
| infiltrate | post-menopausal; advanced |  |
| infiltration | stage=4^bilateral |  |
| malignant cells | bilateral; metastatic |  |
| small cell carcinoma | stage=4^malignant |  |
| retracted nipple | stage=4^lobular |  |
| leiomyosarcoma | non-metastatic |  |
| squamous cell cancer | most likely; metastatic |  |
| malignant disease | metastatic; progressive |  |
| carcinoid | metastatic^span=interval |  |
| liposarcoma | stable; metastatic |  |
| malignant pleural effusion | possible; metastatic |  |
| ulcerative | metastatic; right-sided |  |
| non-small cell carcinoma | metastatic; invasive; lobular |  |
| metaplasia | with contrast; metastatic |  |
| malignant neoplasms | stage=4^metastatic; invasive |  |
| dermatofibrosarcoma | stage=4;c |  |
| rhabdomyosarcoma | primary; metastatic |  |
| carcinoid tumor | stage=4^progressing |  |
| peau d'orange | chronic; metastatic |  |
| malignant cell | stage=n1;m1 |  |
| retraction of nipple | stage=t2;n1;m1 |  |
| cholangiocarcinoma | metastatic; left-sided |  |
| malignant effusion | inflammatory; metastatic |  |
| inflammatory breast disease | stage=4^right-sided |  |
| adenosarcoma | with/without contrast; metastatic |  |
| post lumpectomy pain | stage=4^recurrent; metastatic |  |
| transitional cell carcinoma | stage=4^progressive |  |
| malignant pericardial effusion | stage=4^likely |  |
| carcinoid syndrome | invasive; metastatic |  |

**Note:** The SDS dataset does not contain any term to differentiate between local and distant metastases.
