# Supplementary material for: Risk of seizures in a population of women with BRCA-positive metastatic breast cancer from an electronic health record database in the United States
Source: BMC Cancer. 2023 Jan 24;23:78. doi: 10.1186/s12885-023-10554-6 (PMC9872301; doi:10.1186/s12885-023-10554-6)
Supplement: Supplementary file 3 — Additional file 3: Supplementary Table 3. SDS terms used to identify patients with seizures. [file 12885_2023_10554_MOESM3_ESM.docx]

**Supplementary Table 3.** List of SDS terms used to identify patients with seizures.

| **SDS Term** | **SDS Sentiment** |
| --- | --- |
| seizure | null |
| seizure disorder | have |
| seizure-like activity | diagnose |
| complex partial seizure | present |
| grand mal seizure | positive |
| partial seizure | observe |
| tonic-clonic seizure | develop |
| sgeneralized seizure | exhibit |
| focal seizure |  |
| generalized tonic-clonic seizure |  |
| epileptic seizure |  |
| absence seizure |  |
| simple partial seizure |  |
| petit mal seizure |  |
| tonic seizure |  |
| focal motor seizure |  |
| clonic seizure |  |
| myoclonic seizure |  |
| complex seizure |  |
| partial onset seizure |  |
| atonic seizure |  |
| simple seizure |  |
| partial motor seizure |  |
| simple febrile seizure |  |
| hypomotor seizure |  |
| Jacksonian seizure |  |
| hypermotor seizure |  |
| simple partial onset seizure |  |
| convulsions |  |
| convulsion |  |
| clonic convulsion |  |
