# Supplementary material for: Risk of seizures in a population of women with BRCA-positive metastatic breast cancer from an electronic health record database in the United States
Source: BMC Cancer. 2023 Jan 24;23:78. doi: 10.1186/s12885-023-10554-6 (PMC9872301; doi:10.1186/s12885-023-10554-6)
Supplement: Supplementary file 4 — Additional file 4: Supplementary Table 4. List of anticonvulsants and PARP inhibitors included in the analyses. [file 12885_2023_10554_MOESM4_ESM.docx]

**Supplementary Table 4.** List of anticonvulsants and PARP inhibitors included in the analyses.

| **Anticonvulsant Type** | **Generic name** | **Drug name** |
| --- | --- | --- |
| AMPA receptor antagonists | Perampanel | Fycompa |
| Barbiturates | Mephobarbital | Mebaral |
|  | Phenobarbital | Luminal |
|  | Primidone | Mysoline |
| Benzodiazepines | Clobazam | Onfi (Pro) |
|  | Clobazam | Sympazan (Pro) |
|  | Clonazepam | Klonopin (Pro) |
|  | Clonazepam | Klonopin Wafer |
|  | Diazepam | Diastat (Pro) |
|  | Diazepam | Diastat AcuDial |
|  | Diazepam | Diastat Pediatric |
|  | Diazepam | Diazepam Intensol |
|  | Diazepam | Valium (Pro) |
|  | Diazepam | Valtoco (Pro) |
|  | Diazepam | Zetran |
|  | Lorazepam | Ativan (Pro) |
|  | Lorazepam | Lorazepam Intensol (Pro) |
| Carbamates | Felbamate | Felbatol (Pro) |
| Carbonic anhydrase inhibitors | Acetazolamide | Diamox (Pro) |
|  | Acetazolamide | Diamox Sequels |
|  | Topiramate | Qudexy XR (Pro) |
|  | Topiramate | Topamax (Pro) |
|  | Topiramate | Topamax Sprinkle |
|  | Topiramate | Topiragen (Pro) |
|  | Topiramate | Trokendi XR (Pro) |
|  | Zonisamide | Zonegran (Pro) |
| Dibenzazepines | Carbamazepine | Carbatrol (Pro) |
|  | Carbamazepine | Carnexiv (Pro) |
|  | Carbamazepine | Epitol (Pro) |
|  | Carbamazepine | Equetro (Pro) |
|  | Carbamazepine | Tegretol (Pro) |
|  | Carbamazepine | Tegretol XR |
|  | Eslicarbazepine | Aptiom (Pro) |
|  | Oxcarbazepine | Oxtellar XR (Pro) |
|  | Oxcarbazepine | Trileptal (Pro) |
|  | Rufinamide | Banzel (Pro) |
| Fatty acid derivatives | Divalproex sodium | Depakote (Pro) |
|  | Divalproex sodium | Depakote ER (Pro) |
|  | Divalproex sodium | Depakote Sprinkles (Pro) |
|  | Valproic acid | Depacon (Pro) |
|  | Valproic acid | Depakene (Pro) |
|  | Valproic acid | Stavzor (Pro) |
| Gamma-aminobutyric acid analogs | Gabapentin | Fanatrex |
|  | Gabapentin | Gabarone |
|  | Gabapentin | Gralise (Pro) |
|  | Gabapentin | Neurontin (Pro) |
|  | Gabapentin enacarbil | Horizant (Pro) |
|  | Pregabalin | Lyrica (Pro) |
|  | Pregabalin | Lyrica CR (Pro) |
|  | Vigabatrin | Sabril (Pro) |
| Gamma-aminobutyric acid reuptake inhibitors | Stiripentol | Diacomit (Pro) |
|  | Tiagabine | Gabitril (Pro) |
| Hydantoin anticonvulsants | Ethotoin | Peganone (Pro) |
|  | Fosphenytoin | Cerebyx (Pro) |
|  | Mephenytoin | Mesantoin |
|  | Phenytoin | Dilantin (Pro) |
|  | Phenytoin | Phenytek (Pro) |
|  | Phenytoin | Phenytoin Sodium (Pro) |
|  | Phenytoin | Phenytoin Sodium, Prompt |
| Miscellaneous anticonvulsants | Cannabidiol | Epidiolex (Pro) |
|  | Lacosamide | Vimpat (Pro) |
|  | Magnesium sulfate | Epsom Salt |
| Neuronal potassium channel openers | Ezogabine | Potiga (Pro) |
| Oxazolidinediones | Trimethadione | Tridione (Pro) |
| Pyrrolidines | Brivaracetam | Briviact (Pro) |
|  | Levetiracetam | Elepsia XR (Pro) |
|  | Levetiracetam | Keppra (Pro) |
|  | Levetiracetam | Keppra XR (Pro) |
|  | Levetiracetam | Roweepra (Pro) |
|  | Levetiracetam | Roweepra XR (Pro) |
|  | Levetiracetam | Spritam (Pro) |
| Succinimides | Ethosuximide | Zarontin (Pro) |
|  | Methsuximide | Celontin (Pro) |
| Triazines | Lamotrigine | Lamictal (Pro) |
|  | Lamotrigine | Lamictal CD |
|  | Lamotrigine | Lamictal ODT |
|  | Lamotrigine | Lamictal XR (Pro) |
| **PARP Inhibitors** | | |
| Olaparib | | |
| Talazoparib tosylate | | |
